# Associations between farmers’ market shopping behaviours and objectively measured and self-reported fruit and vegetable intake in a diverse sample of farmers’ market shoppers: a cross-sectional study in New York City and rural North Carolina

**DOI:** 10.1017/S1368980021004602

**Published:** 2022-03

**Authors:** Casey J Kelley, Karla L Hanson, Grace A Marshall, Leah C Volpe, Stephanie Jilcott Pitts, Ann P Rafferty, Rebecca A Seguin-Fowler

**Affiliations:** 1 University of North Carolina at Chapel Hill, School of Medicine, Division of Geriatric Medicine, Center for Aging and Health, 5003 Old Clinic CB#7550, Chapel Hill, NC 27599, USA; 2 Cornell University, Master of Public Health Program, Department of Population Medicine and Diagnostic Sciences, Ithaca, NY, USA; 3 East Carolina University, Brody School of Medicine, Department of Public Health, Greenville, NC, USA; 4 Texas A&M AgriLife Research, Texas A&M University System, College Station, TX, USA

**Keywords:** Farmers’ markets, Shopping behaviours, Fruit and vegetable intake, Skin carotenoids

## Abstract

**Objective:**

To examine cross-sectional associations between farmers’ market shopping behaviours and objectively measured and self-reported fruit and vegetable (FV) intake among rural North Carolina (NC) and New York City (NYC) shoppers.

**Design:**

Cross-sectional intercept surveys were used to assess self-reported FV intake and three measures of farmers’ market shopping behaviour: (1) frequency of purchasing FV; (2) variety of FV purchased and (3) dollars spent on FV. Skin carotenoids, a non-invasive biomarker for FV intake, were objectively measured using pressure-mediated reflection spectroscopy. Associations between farmers’ market shopping behaviours and FV intake were examined using regression models that controlled for demographic variables (e.g. age, sex, race, smoking status, education, income and state).

**Setting:**

Farmers’ markets (*n* 17 markets) in rural NC and NYC.

**Participants:**

A convenience sample of 645 farmers’ market shoppers.

**Results:**

Farmers’ market shoppers in NYC purchased a greater variety of FV and had higher skin carotenoid scores compared with shoppers in rural NC. Among all shoppers, there was a positive, statistically significant association between self-reported frequency of shopping at farmers’ markets and self-reported as well as objectively assessed FV intake. The variety of FV purchased and farmers’ market spending on FV also were positively associated with self-reported FV intake, but not skin carotenoids.

**Conclusion:**

Those who shop for FV more frequently at a farmers’ markets, purchase a greater variety of FV and spend more money on FV have higher self-reported, and in some cases higher objectively measured FV intake. Further research is needed to understand these associations and test causality.

Obesity is a major public health issue in the USA, and its prevalence continues to rise^(1)^. Obesity is linked to greater risk of various cancers, CVD and type 2 diabetes mellitus^(2)^. Inadequate intake of fruits and vegetables (FV) is associated with higher risk of obesity and other diet-related chronic diseases such as heart disease and cancer^(3–8)^. Furthermore, while the variety of FV consumed is known to support good health^(3,8)^, the USA population, on average, consumes few varieties of FV^(3,9)^. Rural populations and racially/ethnically diverse populations have disproportionate rates of chronic diseases and obesity, with low FV intake cited as a contributing factor^(10–12)^.

Evidence suggests that diet-related health disparities may be, in part, due to negative aspects of community food environments^(13,14)^. Access to healthy food sources, such as supermarkets and farmers’ markets, has been inversely associated with obesity, whereas the presence of convenience stores and fast food restaurants has been associated with an increased prevalence of obesity^(13,14)^. Limited evidence suggests that greater access to and use of farmers’ markets are associated with greater self-reported intake of FV^(15–18)^. Additionally, greater overall financial expenditures on FV have been associated with lower mortality^(19)^. In a study of Taiwanese older adults, during a 10-year follow-up period, individuals with the greatest level of FV expenditures, ranking in the fourth and fifth quintiles, had a significantly reduced mortality rate and mortality risk decreased by 12 % and 10 % for every NT $15 (∼$0·50 USD) increase in daily vegetable and fruit expenditures, respectively^(19)^.

Most studies examining associations between shopping at farmers’ markets and FV intake have only used self-reported measures of FV intake, which may be subject to errors in recall, which can increase both random error and systematic bias which tends to overestimate consumption of healthier foods^(20–24)^. Thus, objective measures of FV intake are needed to accurately quantify intake and determine effectiveness of interventions and policies to improve dietary behaviours. Carotenoids are antioxidants that are found in high concentrations in yellow, orange, red and dark green FV, which deposit and accumulate in blood plasma and skin^(25)^. The current criterion standard for objective assessment of FV intake is measurement of carotenoids in blood plasma^(26)^. However, collection, storage and analysis of blood plasma for assessment of carotenoids is invasive, time-consuming and resource-intensive. As an alternative, skin carotenoids measured with pressure-mediated reflection spectroscopy is a validated method to approximate FV intake^(27–29)^.

In this paper, we examined associations between self-reported and objectively measured FV intake and three measures of farmers’ market shopping behaviour: (1) monthly frequency of farmers’ market shopping; (2) variety of FV purchased on one market day and (3) money and/or benefits typically spent on FV at the farmers’ market/week, in both a rural population and a racially/ethnically diverse urban population. We hypothesised that FV intake, measured both by self-report and skin carotenoids, would be positively associated with shopping frequency at farmers’ markets, variety of FV purchased and money and/or benefits spent on FV.

## Methods

### Study design

This cross-sectional study used a convenience sample of farmers’ market shoppers identified through public intercept at ten farmers’ markets in six counties in rural eastern North Carolina (NC) and 7 farmers’ markets in New York City (NYC) from June to August 2019. Eligible participants were at least 18 years of age and able to speak English. The study purpose was explained to each prospective participant, after which they were given the opportunity to ask questions, and verbal consent was obtained by research staff. Participants received a canvas tote bag as compensation.

### Farmers’ market shopping behaviours

Each participant completed a short, self-administered questionnaire (approximately 5–10 min.) electronically on a tablet device. If requested by the participant, the researcher would administer the questionnaire orally. Three farmers’ market shopping behaviours were assessed. Participants were asked ‘*During the farmers’ market season, approximately how often do you purchase fruits or vegetables from the farmers’ market?’* Response options were never, less than once a month, once a month, every other week and once a week. This variable was coded numerically as 0, 0·5, 1, 2 and 4 times/month, respectively. Participants were asked ‘*Which fruits and vegetables did you buy at today’s market?’* Participants responded by selecting from a list of 68 FV, including options to write in any FV that was not included in the list. The total number of FV purchased was used as a measure of FV variety. The questionnaire also asked, ‘*When you go to a farmers’ market, how much money (cash and/or benefits) do you usually spend on fresh fruits and vegetables?’* Benefits were defined as federal food assistance benefits such as the Supplemental Nutrition Assistance Program and participants responded with a numeric value.

### Self-reported fruit and vegetable intake

Self-reported fruit intake was assessed by asking participants *‘How much fruit (in cups) do you eat in an average day?’* Participants were provided examples of quantities of fruit that are approximately equal to one cup and prompted not to include fruit juice. Likewise, vegetable intake was assessed by asking participants *‘How many vegetables (in cups) do you eat in an average day?’* Participants were again provided examples of quantities of vegetables that are approximately equal to one cup and prompted not to include French fries. Response options were whole and half numbers from 0 to 6 cups. These two fruit and vegetable questions were adapted from the American Heart Association’s Life’s Simple 7 score^(30)^.

### Skin carotenoid measurement

Skin carotenoid scores were assessed using pressure-mediated reflection spectroscopy (the ‘Veggie Meter®’, Longevity Link Corporation, Salt Lake City, UT, USA). The Veggie Meter® is a valid, non-invasive, objective approximation of FV intake^(28)^. Participants provided finger scans three times, and the mean of which generated skin carotenoid scores. Skin carotenoid scores range from 0 to 800, with higher numbers indicating higher skin carotenoid levels and thus greater FV intake. This tool has been validated against plasma carotenoids in diverse populations with a correlation between plasma carotenoids and Veggie Meter® assessed skin carotenoid scores of *r* = 0·71, *P* < 0·0001^(28)^. The Veggie Meter® has also been used among diverse New Zealanders with findings indicating positive correlations between skin carotenoid scores and FV intake^(31)^.

### Participant characteristics

Participants reported height in feet and inches and weight in pounds as part of the questionnaire from which BMI was calculated. Participants also reported their age, sex, race, smoking status, education and household income.

### Data analysis

Farmers’ market shoppers were characterised using descriptive statistics, including means and standard deviations for continuous variables and frequencies for categorical variables. Descriptive statistics were calculated for the pooled sample of farmers’ market shoppers and for NYC and NC separately. To test for significant differences between NYC and NC shoppers, *χ* ^2^ tests (for nominal variables), Wilcoxon rank sum tests (for ordinal variables) and *t* tests (for continuous variables) were used.

Linear regression models were used to estimate the unadjusted relationships between each shopping behaviour and each measure of FV intake separately. Then, multivariable regression models were used to control for age, sex, race, smoking status, education, household income and state. To account for clustering of responses within markets, multi-level models included a categorical farmers’ market variable as a random effect when there was enough variation across markets to make this computation possible, which was only the case for skin carotenoids. All models used age < 60 years, male, Caucasian, less than college graduate, household income < $40 000 and NYC as reference groups. Reference groups were chosen based on the median responses to demographic questions. The assumption of non-multicollinearity was tested by analysis of the variance inflation factor and tolerance of each model, and none suggested multicollinearity (variance inflation factor < 10, Tolerance > 0·2). FV purchase variety was not normally distributed (skewness = 3·6, kurtosis = 20·0, Shapiro–Wilk *P* < 0·001) and was log transformed and identical regressions performed as sensitivity analyses. Analysis was performed using SAS version 9.4 (SAS Institute Inc.). The *α* < 0·05 significance level was used to determine statistical significance.

## Results

We surveyed a total of 645 shoppers, in seven farmers’ markets in NYC (*n* 377) and ten farmers’ markets in rural NC (*n* 268). The number of participants surveyed in each market ranged from 1 to 95. Farmers’ market shoppers in this study were mostly female (79·2 %) and more fell into the age 60+ years (36·9 %) category than the other age categories. The shoppers surveyed in NC were relatively homogonous (70·2 % Caucasian, 22·8 % African American and 1·1 % Hispanic/Latino), while those in NYC were significantly more racially and ethnically diverse (23·1 % Caucasian, 30·5 % African American and 33·4 % Hispanic/Latino). Farmers’ market shoppers typically had a household income of at least $40 000 (52·7 %), about half were college graduates (52·6 %), few currently smoked (8·1 %) and mean BMI was 28·0 kg/m^2^. Age, race, ethnicity and BMI differed significantly between shoppers in NYC and rural NC: NC shoppers were older, less racially/ethnically diverse and had higher BMI than NYC shoppers (Table 1).

**Table 1:** Characteristics of farmers’ market shoppers (total *n* 645) in New York City (*n* 377) and rural North Carolina (*n* 268)

	Total (*n* 645)		New York City (*n* 377)		North Carolina (*n* 268)		*P*-value
	Count	%	Count	%	Count	%	
Sex							0·428
Male	124	19·2	67	17·8	57	21·3	
Female	511	79·2	303	80·4	208	77·6	
Other/refused	10	1·6	7	1·9	3	1·1	
Age							**< 0·001**
< 20	13	2·0	8	2·1	5	1·9	
20–29	64	9·9	42	11·1	22	8·2	
30–39	74	11·5	53	14·1	21	7·8	
40–49	100	15·5	66	17·5	34	12·7	
50–59	151	23·4	91	24·1	60	22·4	
60+	238	36·9	112	29·7	126	47·0	
Refused	5	0·8	5	1·3	0	0·0	
Race							**< 0·001**
American Indian	14	2·2	9	2·4	5	1·9	
Asian	28	4·3	28	7·4	0	0·0	
African American	176	27·3	115	30·5	61	22·8	
Native Hawaiian/Pacific Islander	2	0·3	2	0·5	0	0·0	
Caucasian	275	42·6	87	23·1	188	70·2	
Multiracial	55	8·5	46	12·2	9	3·4	
Refused	95	14·7	90	23·9	5	1·9	
Hispanic/Latino							**< 0·001**
Yes	129	20·0	126	33·4	3	1·1	
No	475	73·6	216	57·3	259	96·6	
Refused	41	6·4	35	9·3	6	2·2	
Household income							0·970
< $20 999	88	13·6	69	15·7	29	10·8	
$21 000–$39 999	116	18·0	66	17·5	50	18·7	
$40 000–$59 999	116	18·0	68	18·0	48	17·9	
$60 000–$79 999	65	10·1	34	9·0	31	11·6	
$80 000–$99 999	68	10·5	28	7·4	40	14·9	
> $100 000	91	14·1	50	13·3	41	15·3	
Refused	101	15·7	72	19·1	29	10·8	
Education							0·734
Less than high school graduate	27	4·2	23	6·1	4	1·5	
High school graduate or GED	116	18·0	56	14·9	60	22·4	
Some college	155	24·0	95	25·2	60	22·4	
College graduate	339	52·6	195	51·7	144	53·7	
Refused	8	1·2	8	2·1	0	0·0	
Current smoker							0·900
Yes	52	8·1	30	8·0	22	8·2	
No	579	89·8	338	89·7	241	89·9	
Refused	14	2·2	9	2·4	5	1·9	
BMI (kg/m^2^)							
Mean	28·0		27·5		28·7		**0·035**
SD	6·8		6·6		6·9		

Significance at the *α* < 0·05 level indicated using boldface type.

On average, farmers’ market shoppers purchased FV at the farmers’ market approximately 2·5 times/month, spent $23·15 on FV each visit to the farmers’ market and purchased 2·7 different varieties of FV on the day of survey (Table 2). Compared with farmers’ market shoppers in rural NC, NYC shoppers purchased more varieties of FV (3·1 *v*. 2·3, *P* = 0·011) and spent more on FV at the farmers’ market ($24·88 *v*. $20·87, *P* = 0·035). On average, participants reported consuming 2·2 cups of fruits/d, 2·5 cups of vegetables/d and had a mean skin carotenoid score of 289·2. There was a significant difference in the mean skin carotenoid scores of NYC shoppers and NC shoppers (313·4 *v*. 254·1, *P* < 0·001) but no differences in self-reported fruit or vegetable intake.

**Table 2:** Farmers’ market shopping behaviours and fruit and vegetable intake among farmers’ market shoppers (*n* 645) in New York City and North Carolina

	Total (*n* 645)		New York City (*n* 377)		North Carolina (*n* 268)		*P*-value
	Mean	SD	Mean	SD	Mean	SD	
Farmers’ market shopping behaviours							
Frequency of FV purchase (times/months)	2·5	1·5	2·4	1·5	2·6	1·5	0·069
FV purchase variety (# of different types of FV)	2·7	4·4	3·1	5·3	2·3	2·8	**0·011**
Usual FV purchases ($/week)	23·15	22·75	24·88	21·65	20·87	23·98	**0·035**
Fruit and vegetable intake							
Fruit intake (cups/d)	2·2	1·4	2·2	1·4	2·1	1·3	0·082
Vegetable intake (cups/d)	2·5	1·4	2·4	1·4	2·5	1·3	0·328
Mean skin carotenoid score	289·2	131·2	313·4	140·6	254·1	107·2	**< 0·001**

FV, fruits and vegetables.

Significance at the *α* < 0·05 level indicated using boldface type.

There were positive, statistically significant associations between FV purchasing frequency (times/months) and self-reported fruit (*P* = 0·007) and vegetable intake (*P* < 0·001) and mean skin carotenoid scores (*P* = 0·009) in the adjusted models (Table 3). FV purchase variety was also positively associated with self-reported fruit intake (*P* = 0·002) and self-reported vegetable intake (*P* = 0·005), but not skin carotenoids, in the multivariate regression models. Sensitivity analyses using the log of FV variety produced results that were similar in direction and significance (data not shown). After adjustment, the amount of money typically spent on FV at a farmers’ market was positively associated with self-reported fruit intake (*P* < 0·001) and self-reported vegetable intake (*P* < 0·001), but not skin carotenoids. Adjustment for state was significant in models of objectively measured carotenoids, but not self-reported FV intake.

**Table 3:** Associations between farmers’ market shopping behaviours and fruit and vegetable intake among farmers’ market shoppers (*n* 645)

	Self-reported fruit intake			Self-reported vegetable intake			Mean skin carotenoid score*		
	*n*	Estimate	*P*-value	*n*	Estimate	*P*-value	*n*	Estimate	*P*-value
Frequency of FV purchases (times/months)									
Unadjusted	635	0·09	0·010	636	0·14	< 0·001	611	4·85	0·164
+Adjustment for	527	0·11	0·007	528	0·15	< 0·001	501	9·83	0·009
Age (60+ years)		0·04	0·738		0·10	0·417		−3·80	0·748
Female		−0·00	0·982		−0·16	0·269		−30·17	0·028
Race									
African American		0·17	0·238		0·24	0·088		18·77	0·159
Other/multi-race		0·01	0·959		−0·09	0·614		3·42	0·833
Current smoker		−0·03	0·905		0·03	0·878		−66·08	< 0·001
Education (College graduate)		−0·07	0·606		0·04	0·753		42·13	< 0·001
Household income (≥ $40 000)		0·08	0·559		0·21	0·097		16·91	0·170
State (North Carolina)		−0·20	0·116		0·12	0·310		−59·32	< 0·001
FV purchase variety (# of types of FV)									
Unadjusted	636	0·03	0·006	637	0·03	0·016	612	0·23	0·846
+Adjustment for	528	0·04	0·002	529	0·04	0·005	502	0·26	0·842
Age (60+ years)		0·08	0·498		0·17	0·165		2·72	0·816
Female		−0·04	0·764		−0·20	0·174		−31·21	0·024
Race									
African American		0·17	0·235		0·23	0·104		17·63	0·186
Other/multi-race		0·00	0·996		−0·10	0·545		2·78	0·864
Current smoker		−0·04	0·855		−0·00	0·992		−70·02	< 0·001
Education (College graduate)		−0·09	0·500		0·01	0·961		39·71	< 0·001
Household income (≥ $40 000)		0·08	0·545		0·20	0·120		14·61	0·238
State (North Carolina)		−0·15	0·244		0·17	0·166		−57·16	< 0·001
Usual FV purchases ($/week)									
Unadjusted	576	0·01	< 0·001	576	0·01	< 0·001	553	0·23	0·344
+Adjustment for	485	0·01	< 0·001	486	0·01	< 0·001	462	0·15	0·529
Age (60+ years)		0·11	0·386		0·18	0·148		6·88	0·576
Female		−0·01	0·931		−0·18	0·231		−30·20	0·037
Race									
African American		0·18	0·215		0·18	0·202		15·52	0·273
Other/multi-race		0·02	0·895		−0·13	0·469		1·52	0·930
Current smoker		−0·09	0·705		−0·03	0·903		−76·93	< 0·001
Education (College graduate)		−0·11	0·419		−0·02	0·864		37·37	0·003
Household income (≥ $40 000)		0·06	0·635		0·21	0·105		18·66	0·152
State (North Carolina)		−0·12	0·371		0·18	0·171		−54·52	< 0·001

FV, fruits and vegetables.

Significance at the *α* < 0·05 level indicated using boldface type.

* Farmers’ market was included as a random effect in this model to account for clustering.

## Discussion

In the current study, we found that frequency of shopping at farmers’ markets was positively associated with self-reported and objectively assessed FV intake in a diverse sample of farmers’ markets shoppers from two geographic areas – one urban (NYC) and one rural (NC). This is in agreement with prior studies which found that increased shopping at farmers’ markets is associated with greater FV intake^(15–18)^, yet adds to the current literature by demonstrating this cross-sectional association persists when FV intake is assessed by a valid, objective measure (skin carotenoids). We also found that the amount of money typically spent on FV purchases on each trip to the farmers’ market and the variety of FV purchased at the farmers’ market on the day of survey were positively associated with self-reported FV intake, but neither was associated with skin carotenoids. These seemingly contradictory findings may be because many of the vegetables sold at farmers’ markets, such as cucumbers, squash, onions and potatoes are low in carotenoids^(32)^.

Our findings support the evidence that frequent farmers’ market shopping is associated with greater FV intake and adds data regarding two other relevant dimensions of the farmers’ market shopping experience (amount of money spent on FV at the farmers’ market and variety of FV purchased). Our findings are in agreement with others that have found that greater vegetable variety is associated with higher intake of vegetables^(33–35)^.

This study also adds to the literature regarding expenditures at farmers’ markets: a Canadian study revealed that farmers’ market shoppers spent on average $5 CAD (∼$3·60 USD)/trip to the market *v*. $23/trip in our sample^(36)^. The mean variety of FV purchased in the Canadian study was similar to the mean variety in our study (2·8 *v*. 2·7)^(36)^. In addition, Freedman *et al*.^(37)^ suggested that an approach that includes the establishment of farmers’ markets in low-income neighbourhoods, acceptance of federal food assistance benefits for payment and availability of healthy food incentive programming may increase purchasing at farmers’ markets among underserved populations. In the current paper, we examined cross-sectional associations between farmers’ market shopping behaviours and FV intake. We hypothesised that more intense shopping behaviours (greater frequency, variety and monetary value of FV purchased at farmers’ markets) would be positively associated with FV intake. This hypothesis was supported, suggesting that research should test the relative effectiveness of programmes to increase farmers’ market shopping intensity (e.g. double-up bucks, variety incentives, return visitor incentives) in addition to mechanisms that simply incentivise initial farmers’ market attendance (e.g. first time shopper coupons, introduction coupons).

Interestingly, there were significant state-level differences in mean skin carotenoid scores, an objective measure of FV intake, with urban NYC shoppers having higher skin carotenoids than rural NC shoppers. This is in agreement with prior studies demonstrating differences in FV intake by rural/urban residence^(10,11)^ and should be examined in future studies.

Much of the literature on farmers’ market shopping is among Caucasian, higher socio-economic status, females^(38–40)^. The geographic and racial/ethnic variability of the sample is a key strength of this study, along with the assessment of three dimensions of farmers’ market shopping behaviour, and the use of an objective measure of FV intake (skin carotenoids) in addition to self-reported FV intake data. However, the study was limited by its cross-sectional design from which we could not infer causality between farmers’ market shopping behaviours and FV intake nor understand the direction of any potential links. Another limitation of this study is the possibility of measurement bias in self-reported data. Farmers’ market shopping behaviours and FV intake may be subject to potential overestimation of positive behaviour due to social desirability bias. Assessment of FV intake used questions from the widely employed American Heart Association’s Life’s Simple 7 questionnaire^(30)^ to reduce this potential for bias. It is also possible that skin carotenoid scores were influenced by environmental factors. Smoking status is known to be associated with skin carotenoids and was controlled for in adjusted models, but other environmental factors such as secondhand smoke in the home were not available^(41)^. Additionally, the measure of FV variety was not divided into smaller groupings of FV, some of which may be carotenoid rich, while others are not a significant source of carotenoids. This may have confounded potential associations between FV variety and skin carotenoids. Finally, generalisability is limited based upon the use of convenience samples in rural NC and urban NYC.

## Conclusion

The current study contributes important findings related to shopping behaviours and spending at farmers’ markets and their positive relationships with FV intake among shoppers. Further research is needed to test causality between farmers’ market shopping behaviours and FV intake and to test the relative effectiveness of programs to increase farmers’ market shopping intensity in addition to farmers’ market introductions, in order to effectively promote FV intake in a variety of community food environments.

## Supplementary material

For supplementary material accompanying this paper visit https://doi.org/10.1017/S1368980021004602. click here to view supplementary material
